# Supplementary material for: Evaluation of the Conversations about Non-Suicidal Self-Injury Mental Health First Aid Course: Effects on Knowledge, Stigmatising Attitudes, Confidence and Helping Behaviour
Source: Int J Environ Res Public Health. 2023 Feb 20;20(4):3749. doi: 10.3390/ijerph20043749 (PMC9963380; doi:10.3390/ijerph20043749)
Supplement: Supplementary file 1 [file ijerph-20-03749-s001.zip › S1 Copies of the pre-, post- and six-month follow-up surveys.pdf]

Evaluating the Mental Health First Aid Course for Non-Suicidal Self-Injury

(T1)

Information about you

Please provide us with your name, phone number and email address. (We ask for this information so that we can match your three surveys and send you the final survey. Once your surveys are matched, your name is deleted from the information in the survey. Only the research officers have access to identifying information and ***we will not share your information with anyone***.)

1. Name

2. Email *(If you would prefer we send you a paper copy of the 6-month follow-up survey please give us your postal address.)*

3. Phone number

4. What is your gender?

- ☐ Male
- ☐ Female
- ☐ I prefer to self-describe (please state)

5. What is your age? (You must be 18 or older to participate in this research.)

6. What is the highest level of education you have completed?

- ☐ Year 9 or lower
- ☐ Year 10, 11, or 12
- ☐ Certificate, Trade or Apprenticeship
- ☐ University
- ☐ Other, please specify:

7. Are you:

- ☐ Aboriginal
- ☐ Torres Strait Islander
- ☐ Both
- ☐ Neither

8. What is the post code of where you live? ☐

9. Do you speak a language other than English as your first language at home?

- ☐ No
- ☐ Yes

10. Have you done any previous mental health training (including short courses or professional training)? If yes, what have you done:

- ☐ No
- ☐ Yes, please specify:

11. Have you done any previous training about non-suicidal self-injury? (Non-suicidal self-injury refers to any self-inflicted injury that is not intended to result in death.)

- ☐ No
- ☐ Yes, please specify:

12. Do you have any personal or professional experience with non-suicidal self-injury? *Please tick all that apply.*

☐ No personal or professional experience

☐ Yes, in myself

☐ Yes, in clients/patients

☐ Yes, in a friend/s

☐ Yes, in a student

☐ Yes, in my family

☐ Yes, in a colleague

☐ Yes, in an acquaintance

☐ Other, please specify:

☐ I'd rather not say

13. Why are you interested in doing this course? *Please tick all that apply.*

☐ As part of continuing education for my workplace/profession

☐ As part of training for a volunteer job

☐ I currently support/know someone who engages in non-suicidal self-injury

☐ In the past, I have had contact with someone who has engaged in non-suicidal self-injury

☐ I have engaged in non-suicidal self-injury

☐ Other, please specify:

What would you do?

The following section concerns a hypothetical person called Alicia. *Imagine she is someone you know and care about.*

Alicia is your 18 year old niece who has recently completed her final year of high school and is awaiting university offers. Her parents have recently separated after several years of fighting and bitterness, which has taken a toll on Alicia. She often spends time at your house to get away from the difficulties at home. In the last year you have noticed bruising and scratches on her arms and legs, but when questioned Alicia says that she is clumsy and falls over all the time. Alicia usually loves to spend time in your backyard pool, however lately she doesn't want to swim even though it has been unseasonably hot. In fact, you've noticed she has been wearing her winter clothes in spite of the heat. Today you noticed some cuts on her arm.

14. How confident are you in your ability to help Alicia? (Circle the number that represents your answer.)

1	2	3	4	5
Not at all confident	A little bit confident	Moderately confident	Quite a bit confident	Extremely confident

15. How likely would you be to do the following to help Alicia:

	Very unlikely	Unlikely	Neither unlikely or likely	Likely	Very likely
Wait and see if her problems go away.					
Wait and see if her problems get worse.					
Wait and see if Alicia says that she thinks she might have a problem.					
Directly express your concerns to Alicia about her injuries.					
Ignore Alicia's injuries because she could be doing this to get attention.					
Ask Alicia about why she is injuring herself.					
Ask Alicia about her feelings that have led her to injure herself.					
Let Alicia know how distressing her injuries are to you.					
Tell Alicia to stop injuring herself.					
Tell Alicia that if she continues to injure herself she will have life-long scars.					

15. How likely would you be to do the following to help Alicia (CONT.):

	Very unlikely	Unlikely	Neither unlikely or likely	Likely	Very likely
Ask Alicia if she is having thoughts of suicide.					
Tell Alicia that her self-injuring is making things worse for her parents who are going through a difficult time.					
Help Alicia find ways to make her life more manageable or reduce her distress.					
Tell Alicia she can call you when she is feeling like injuring herself.					
Promise Alicia that if she stops injuring herself you will reward her, e.g. go shopping and buy her something.					
Tell Alicia that there are sources of help and support available.					
Offer to help Alicia to seek mental health treatment.					
Ask Alicia if there are things she can do that will help her delay injuring herself.					

16. The next few questions contain statements about Alicia's problem. (Please indicate how strongly you agree or disagree with each statement.)

	Strongly disagree	Disagree	Neither agree nor disagree	Agree	Strongly Agree
Alicia could snap out of it if she wanted.					
Alicia's problem is a sign of personal weakness.					
Alicia's problem is not a real medical illness.					
Alicia is dangerous (to others).					
It is best to avoid Alicia so that you don't develop this problem yourself.					
Alicia's problem makes her unpredictable.					
I would not tell anyone if I had a problem like Alicia's.					

17. The following questions ask how you would feel about spending time with *__a person with a problem like__* Alicia's. Would you be happy to...

	**Definitely not**	**Probably not**	**Not sure**	**Yes, probably**	**Yes, definitely**
Move next door to them?					
Spend an evening socialising with them?					
Make friends with them?					
Work closely with them on a project?					

17. The following questions ask how you would feel about spending time with *__a person with a problem like__* Alicia's. Would you be happy to... (CONT.)

	**Definitely not**	**Probably not**	**Not sure**	**Yes, probably**	**Yes, definitely**
Have them marry into your family?					
Employ them?					
Vote for a politician if you knew they had suffered a problem like Alicia's?					

__Opinions about non-suicidal self-injury__

18. The next section contains statements about non-suicidal self-injury. Please indicate whether you agree or disagree with each statement, or if you don't know.

Tattoos and piercings are a form of non-suicidal self-injury.	DISAGREE	AGREE	DON'T KNOW
A person engaging in non-suicidal self-injury is likely to have anxiety or depression.	DISAGREE	AGREE	DON'T KNOW
It is best to try to determine the reasons behind the non-suicidal self-injury before approaching the person about it.	DISAGREE	AGREE	DON'T KNOW
Non-suicidal self-injury is a coping mechanism.	DISAGREE	AGREE	DON'T KNOW
You should only approach a person about non-suicidal self-injury if the injuries are serious.	DISAGREE	AGREE	DON'T KNOW

18. The next section contains statements about non-suicidal self-injury (CONT.).

The most common method of non-suicidal self-injury is burning the skin.	DISAGREE	AGREE	DON'T KNOW
Non-suicidal self-injury rarely occurs in males.	DISAGREE	AGREE	DON'T KNOW
You should ignore a person's non-suicidal self-injury so as not to reinforce the behaviour.	DISAGREE	AGREE	DON'T KNOW
For some people, non-suicidal self-injury is a way to ask for help.	DISAGREE	AGREE	DON'T KNOW
Frequent unexplained injuries may indicate that a person is engaging in non-suicidal self-injury.	DISAGREE	AGREE	DON'T KNOW
Non-suicidal self-injury can help a person feel better.	DISAGREE	AGREE	DON'T KNOW
People can sometimes engage in non-suicidal self-injury as a way to reduce suicidal feelings.	DISAGREE	AGREE	DON'T KNOW
If a person has harmed themselves by taking an overdose of medication or consuming a substance that might be poisonous, you should wait and see if they show symptoms of being ill before calling an ambulance.	DISAGREE	AGREE	DON'T KNOW
Professional medical attention is required for any non-suicidal self-injury regardless of severity.	DISAGREE	AGREE	DON'T KNOW

18. The next section contains statements about non-suicidal self-injury (CONT.).

A person who engages in non-suicidal self-injury needs someone to take charge and tell them what to do in order to cope with their urges to self-injure.	DISAGREE	AGREE	DON'T KNOW
If the person is receiving mental health care, you should ask them if their treating professional knows about their injuries.	DISAGREE	AGREE	DON'T KNOW

People you have been concerned about

19. In the past 6 months, have you had contact with someone who you thought might be engaging in non-suicidal self-injury?

☐ No ***(you do not need to complete any more of the survey)***

☐ Yes, one person ***(please continue with the next question)***

☐ Yes, two or more people ***(please continue with the next question)***

☐ I'd rather not say ***(you do not need to complete any more of the survey)***

If you had contact with more than one person over the past 6 months, think about the person you had the most contact with.

20. What is the age of the person?

☐ under 10

☐ 10-17

☐ 18-24

☐ 25-34

☐ 35-54

☐ 55-74

☐ 75+

21. What is their gender:

☐ Male

☐ Female

☐ Other

☐ Unsure

22.What is their relationship to you?

☐ Family member

☐ Friend

☐ Work colleague

☐ Acquaintance

☐ Stranger

☐ Client/patient

☐ Student

☐ Other, please specify:

23. Did you do any of the following to help the person?

☐ Waited to see if their problems went away.

☐ Waited to see if their problems got worse.

☐ Waited to see if they said that they thought they might have a problem.

☐ Directly expressed your concerns to them about their injuries.

☐ Ignored their injuries because you thought they could be doing it to get attention.

☐ Asked them about why they were injuring themselves.

☐ Asked them about their feelings that have led them to injure themselves.

☐ Let them know how distressing their injuries were to you.

☐ Told them to stop injuring themselves.

23. Did you do any of the following to help the person (CONT.)?

☐ Told them that if they continued to injure themselves they will have life-long scars.

☐ Asked them if they were having thoughts of suicide.

☐ Told them that their self-injuring is difficult for the people around them.

☐ Helped them find ways to make their life more manageable or reduce their distress.

☐ Told them they could call you when they were feeling like injuring themselves.

☐ Promised them that if they stopped injuring themselves you would reward them, e.g. go shopping and buy them something.

☐ Told them that there are sources of help and support available.

☐ Offered to help them seek mental health treatment.

☐ Asked them if there were things you could do that would help them delay injuring themselves.

☐ You did not do anything ***(please go to question 24 on this page).***

☐ You did something else. Please let us know what you did:

24. How confident were you in your ability to help the person?

1<br>Not at all confident	2<br>A little bit confident	3<br>Moderately confident	4<br>Quite a bit confident	5<br>Extremely confident

25. *If you DID try to help the person*, what were the effects on the person of what you did?

26. *If you DID try to help the person*, what did the person do as a result of your help?

27. *If you DIDN'T try to help the person*, are there any particular reasons that you did not try to help? If so, please describe these reasons.

That is the end of the survey. Thank you for your time.

Evaluating the Mental Health First Aid Course for Non-Suicidal Self-Injury

(T2)

Please provide us with your name, phone number and email address. (We ask for this information so that we can match your three surveys and send you the final survey. Once your surveys are matched, your name is deleted from the information in the survey. Only the research officers have access to identifying information and ***we will not share your information with anyone***.)

1. Name

2. Email *(If you would prefer we send you a paper copy of the 6-month follow-up survey please give us your postal address.)*

3. Phone number

What would you do?

The following section concerns a hypothetical person called Alicia. *Imagine she is someone you know and care about.*

Alicia is your 18 year old niece who has recently completed her final year of high school and is awaiting university offers. Her parents have recently separated after several years of fighting and bitterness, which has taken a toll on Alicia. She often spends time at your house to get away from the difficulties at home. In the last year you have noticed bruising and scratches on her arms and legs, but when questioned Alicia says that she is clumsy and falls over all the time. Alicia usually loves to spend time in your backyard pool, however lately she doesn't want to swim even though it has been unseasonably hot. In fact, you've noticed she has been wearing her winter clothes in spite of the heat. Today you noticed some cuts on her arm.

4. How confident are you in your ability to help Alicia? (Circle the number that represents your answer.)

1<br>Not at all confident	2<br>A little bit confident	3<br>Moderately confident	4<br>Quite a bit confident	5<br>Extremely confident

5. How likely would you be to do the following to help Alicia:

	**Very unlikely**	**Unlikely**	**Neither unlikely or likely**	**Likely**	**Very likely**
Wait and see if her problems go away.					
Wait and see if her problems get worse.					
Wait and see if Alicia says that she thinks she might have a problem.					
Directly express your concerns to Alicia about her injuries.					
Ignore Alicia's injuries because she could be doing this to get attention.					
Ask Alicia about why she is injuring herself.					
Ask Alicia about her feelings that have led her to injure herself.					

5. How likely would you be to do the following to help Alicia (CONT.):

	Very unlikely	Unlikely	Neither unlikely or likely	Likely	Very likely
Let Alicia know how distressing her injuries are to you.					
Tell Alicia to stop injuring herself.					
Tell Alicia that if she continues to injure herself she will have life-long scars.					
Ask Alicia if she is having thoughts of suicide.					
Tell Alicia that her self-injuring is making things worse for her parents who are going through a difficult time.					
Help Alicia find ways to make her life more manageable or reduce her distress.					
Tell Alicia she can call you when she is feeling like injuring herself.					
Promise Alicia that if she stops injuring herself you will reward her, e.g. go shopping and buy her something.					

5. How likely would you be to do the following to help Alicia (CONT.):

	**Very unlikely**	**Unlikely**	**Neither unlikely or likely**	**Likely**	**Very likely**
Tell Alicia that there are sources of help and support available.					
Offer to help Alicia to seek mental health treatment.					
Ask Alicia if there are things she can do that will help her delay injuring herself.					

6. The next few questions contain statements about Alicia's problem. (Please indicate how strongly you agree or disagree with each statement.)

	Strongly disagree	Disagree	Neither agree nor disagree	Agree	Strongly Agree
Alicia could snap out of it if she wanted.					
Alicia's problem is a sign of personal weakness.					
Alicia's problem is not a real medical illness.					
Alicia is dangerous (to others).					
It is best to avoid Alicia so that you don't develop this problem yourself.					
Alicia's problem makes her unpredictable.					
I would not tell anyone if I had a problem like Alicia's.					

7. The following questions ask how you would feel about spending time with *__a person with a problem like__* Alicia's. Would you be happy to...

	**Definitely not**	**Probably not**	**Not sure**	**Yes, probably**	**Yes, definitely**
Move next door to them?					
Spend an evening socialising with them?					
Make friends with them?					
Work closely with them on a project?					
Have them marry into your family?					
Employ them?					
Vote for a politician if you knew they had suffered a problem like Alicia's?					

Opinions about non-suicidal self-injury

8. The next section contains statements about non-suicidal self-injury. Please indicate whether you agree or disagree with each statement, or if you don’t know.

Tattoos and piercings are a form of non-suicidal self-injury.	DISAGREE	AGREE	DON’T KNOW
A person engaging in non-suicidal self-injury is likely to have anxiety or depression.	DISAGREE	AGREE	DON’T KNOW
It is best to try to determine the reasons behind the non-suicidal self-injury before approaching the person about it.	DISAGREE	AGREE	DON’T KNOW
Non-suicidal self-injury is a coping mechanism.	DISAGREE	AGREE	DON’T KNOW
You should only approach a person about non-suicidal self-injury if the injuries are serious.	DISAGREE	AGREE	DON’T KNOW
The most common method of non-suicidal self-injury is burning the skin.	DISAGREE	AGREE	DON’T KNOW
Non-suicidal self-injury rarely occurs in males.	DISAGREE	AGREE	DON’T KNOW
You should ignore a person’s non-suicidal self-injury so as not to reinforce the behaviour.	DISAGREE	AGREE	DON’T KNOW
For some people, non-suicidal self-injury is a way to ask for help.	DISAGREE	AGREE	DON’T KNOW
Frequent unexplained injuries may indicate that a person is engaging in non-suicidal self-injury.	DISAGREE	AGREE	DON’T KNOW

8. The next section contains statements about non-suicidal self-injury (CONT.).

Non-suicidal self-injury can help a person feel better.	DISAGREE	AGREE	DON'T KNOW
People can sometimes engage in non-suicidal self-injury as a way to reduce suicidal feelings.	DISAGREE	AGREE	DON'T KNOW
If a person has harmed themselves by taking an overdose of medication or consuming a substance that might be poisonous, you should wait and see if they show symptoms of being ill before calling an ambulance.	DISAGREE	AGREE	DON'T KNOW
Professional medical attention is required for any non-suicidal self-injury regardless of severity.	DISAGREE	AGREE	DON'T KNOW
A person who engages in non-suicidal self-injury needs someone to take charge and tell them what to do in order to cope with their urges to self-injure.	DISAGREE	AGREE	DON'T KNOW
If the person is receiving mental health care, you should ask them if their treating professional knows about their injuries.	DISAGREE	AGREE	DON'T KNOW

Course satisfaction

9. How new was the information in the course to you?

Not at all new		Somewhat new		Mostly new
☐	☐	☐	☐	☐

10. How much of the information in the program did you understand?

None of it		Some of it		Most of it
☐	☐	☐	☐	☐

11. How well was the program presented?

Very poorly		Somewhat well		Very well
☐	☐	☐	☐	☐

12. How relevant was the content for you?

Not very much		Somewhat		Very much
☐	☐	☐	☐	☐

13. Please rate how much you liked the following parts of the program:

	Not very much		Somewhat		Very much
Handbook	☐	☐	☐	☐	☐
Powerpoint slides	☐	☐	☐	☐	☐
Videos	☐	☐	☐	☐	☐
Activities	☐	☐	☐	☐	☐

14. What aspects of the course did you find most helpful?

15. Is there anything in the course that could be improved?

16. Is there anything else you would like to tell us about the course?

Please take this page with you

Thank you for completing this survey!

If taking this survey brings up difficult emotions, you may wish to contact **Lifeline on 13 11 14** or **Suicide Call Back Service on 1300 659 467** for free online and telephone counselling, available 24 hours a day, seven days a week.

Please also contact the Research Officer, Kathy Bond on tel: 03 9079 0207 or email: kathybond@mhfa.com.au so that we can know how many experience distress.

Evaluating the Mental Health First Aid Course for Non-Suicidal Self-Injury

(T3)

Please provide us with your name, phone number and email address. (We ask for this information so that we can match your three surveys and send you the final survey. Once your surveys are matched, your name is deleted from the information in the survey. Only the research officers have access to identifying information and ***we will not share your information with anyone***.)

1. Name

2. Email

3. Phone number

What would you do?

The following section concerns a hypothetical person called Alicia. *Imagine she is someone you know and care about.*

Alicia is your 18 year old niece who has recently completed her final year of high school and is awaiting university offers. Her parents have recently separated after several years of fighting and bitterness, which has taken a toll on Alicia. She often spends time at your house to get away from the difficulties at home. In the last year you have noticed bruising and scratches on her arms and legs, but when questioned Alicia says that she is clumsy and falls over all the time. Alicia usually loves to spend time in your backyard pool, however lately she doesn't want to swim even though it has been unseasonably hot. In fact, you've noticed she has been wearing her winter clothes in spite of the heat. Today you noticed some cuts on her arm.

4. How confident are you in your ability to help Alicia? (Circle the number that represents your answer.)

1<br>Not at all confident	2<br>A little bit confident	3<br>Moderately confident	4<br>Quite a bit confident	5<br>Extremely confident

5. How likely would you be to do the following to help Alicia:

	**Very unlikely**	**Unlikely**	**Neither unlikely or likely**	**Likely**	**Very likely**
Wait and see if her problems go away.					
Wait and see if her problems get worse.					
Wait and see if Alicia says that she thinks she might have a problem.					
Directly express your concerns to Alicia about her injuries.					
Ignore Alicia's injuries because she could be doing this to get attention.					
Ask Alicia about why she is injuring herself.					
Ask Alicia about her feelings that have led her to injure herself.					

5. How likely would you be to do the following to help Alicia (CONT.):

	Very unlikely	Unlikely	Neither unlikely or likely	Likely	Very likely
Let Alicia know how distressing her injuries are to you.					
Tell Alicia to stop injuring herself.					
Tell Alicia that if she continues to injure herself she will have life-long scars.					
Ask Alicia if she is having thoughts of suicide.					
Tell Alicia that her self-injuring is making things worse for her parents who are going through a difficult time.					
Help Alicia find ways to make her life more manageable or reduce her distress.					
Tell Alicia she can call you when she is feeling like injuring herself.					
Promise Alicia that if she stops injuring herself you will reward her, e.g. go shopping and buy her something.					

5. How likely would you be to do the following to help Alicia (CONT.):

	**Very unlikely**	**Unlikely**	**Neither unlikely or likely**	**Likely**	**Very likely**
Tell Alicia that there are sources of help and support available.					
Offer to help Alicia to seek mental health treatment.					
Ask Alicia if there are things she can do that will help her delay injuring herself.					

6. The next few questions contain statements about Alicia's problem. (Please indicate how strongly you agree or disagree with each statement.)

	Strongly disagree	Disagree	Neither agree nor disagree	Agree	Strongly Agree
Alicia could snap out of it if she wanted.					
Alicia's problem is a sign of personal weakness.					
Alicia's problem is not a real medical illness.					
Alicia is dangerous (to others).					
It is best to avoid Alicia so that you don't develop this problem yourself.					
Alicia's problem makes her unpredictable.					
I would not tell anyone if I had a problem like Alicia's.					

7. The following questions ask how you would feel about spending time with *__a person with a problem like__* Alicia's. Would you be happy to...

	**Definitely not**	**Probably not**	**Not sure**	**Yes, probably**	**Yes, definitely**
Move next door to them?					
Spend an evening socialising with them?					
Make friends with them?					
Work closely with them on a project?					
Have them marry into your family?					
Employ them?					
Vote for a politician if you knew they had suffered a problem like Alicia's?					

Opinions about non-suicidal self-injury

8. The next section contains statements about non-suicidal self-injury. Please indicate whether you agree or disagree with each statement, or if you don't know.

Tattoos and piercings are a form of non-suicidal self-injury.	DISAGREE	AGREE	DON'T KNOW
A person engaging in non-suicidal self-injury is likely to have anxiety or depression.	DISAGREE	AGREE	DON'T KNOW
It is best to try to determine the reasons behind the non-suicidal self-injury before approaching the person about it.	DISAGREE	AGREE	DON'T KNOW
Non-suicidal self-injury is a coping mechanism.	DISAGREE	AGREE	DON'T KNOW
You should only approach a person about non-suicidal self-injury if the injuries are serious.	DISAGREE	AGREE	DON'T KNOW
The most common method of non-suicidal self-injury is burning the skin.	DISAGREE	AGREE	DON'T KNOW
Non-suicidal self-injury rarely occurs in males.	DISAGREE	AGREE	DON'T KNOW
You should ignore a person's non-suicidal self-injury so as not to reinforce the behaviour.	DISAGREE	AGREE	DON'T KNOW
For some people, non-suicidal self-injury is a way to ask for help.	DISAGREE	AGREE	DON'T KNOW
Frequent unexplained injuries may indicate that a person is engaging in non-suicidal self-injury.	DISAGREE	AGREE	DON'T KNOW

8. The next section contains statements about non-suicidal self-injury (CONT.).

Non-suicidal self-injury can help a person feel better.	DISAGREE	AGREE	DON'T KNOW
People can sometimes engage in non-suicidal self-injury as a way to reduce suicidal feelings.	DISAGREE	AGREE	DON'T KNOW
If a person has harmed themselves by taking an overdose of medication or consuming a substance that might be poisonous, you should wait and see if they show symptoms of being ill before calling an ambulance.	DISAGREE	AGREE	DON'T KNOW
Professional medical attention is required for any non-suicidal self-injury regardless of severity.	DISAGREE	AGREE	DON'T KNOW
A person who engages in non-suicidal self-injury needs someone to take charge and tell them what to do in order to cope with their urges to self-injure.	DISAGREE	AGREE	DON'T KNOW
If the person is receiving mental health care, you should ask them if their treating professional knows about their injuries.	DISAGREE	AGREE	DON'T KNOW

People you have been concerned about

9. In the past 6 months, have you had contact with someone who you thought might be engaging in non-suicidal self-injury?

☐ No ***(you do not need to complete any more of the survey)***

☐ Yes, one person ***(please continue with the next question)***

☐ Yes, two or more people ***(please continue with the next question)***

☐ I'd rather not say ***(you do not need to complete any more of the survey)***

If you had contact with more than one person over the past 6 months, <u>think about the person you had the most contact with.</u>

10. What is the age of the person?

☐ under 10

☐ 10-17

☐ 18-24

☐ 25-34

☐ 35-54

☐ 55-74

☐ 75+

11. What is their gender:

☐ Male

☐ Female

☐ Other

☐ Unsure

12.What is their relationship to you?

☐ Family member

☐ Friend

☐ Work colleague

☐ Acquaintance

☐ Stranger

☐ Client/patient

☐ Student

☐ Other, please specify:

13. Did you do any of the following to help the person?

☐ Waited to see if their problems went away.

☐ Waited to see if their problems got worse.

☐ Waited to see if they said that they thought they might have a problem.

☐ Directly expressed your concerns to them about their injuries.

☐ Ignored their injuries because you thought they could be doing it to get attention.

☐ Asked them about why they were injuring themselves.

☐ Asked them about their feelings that have led them to injure themselves.

☐ Let them know how distressing their injuries were to you.

☐ Told them to stop injuring themselves.

☐ Told them that if they continued to injure themselves they will have life-long scars.

☐ Asked them if they were having thoughts of suicide.

☐ Told them that their self-injuring is difficult for the people around them.

☐ Helped them find ways to make their life more manageable or reduce their distress.

☐ Told them they could call you when they were feeling like injuring themselves.

☐ Promised them that if they stopped injuring themselves you would reward them, e.g. go shopping and buy them something.

☐ Told them that there are sources of help and support available.

☐ Offered to help them seek mental health treatment.

☐ Asked them if there were things you could do that would help them delay injuring themselves.

☐ You did not do anything ***(please go to question 24 on this page).***

☐ You did something else. Please let us know what you did:

14. How confident were you in your ability to help the person?

1<br>Not at all confident	2<br>A little bit confident	3<br>Moderately confident	4<br>Quite a bit confident	5<br>Extremely confident

15. *<u>If you DID try to help the person</u>*, what were the effects on the person of what you did?

16. *<u>If you DID try to help the person</u>*, what did the person do as a result of your help?

17. *<u>If you DIDN'T try to help the person</u>*, are there any particular reasons that you did not try to help? If so, please describe these reasons.

Please take this page with you

Thank you for completing this survey!

If taking this survey brings up difficult emotions, you may wish to contact **Lifeline on 13 11 14** or **Suicide Call Back Service on 1300 659 467** for free online and telephone counselling, available 24 hours a day, seven days a week.

Please also contact the Research Officer, Kathy Bond on tel: 03 9079 0207 or email: kathybond@mhfa.com.au so that we can keep a log of any adverse events.

Mental Health First Aid for Non-Suicidal Self-Injury: An evaluation

Consent form for persons participating in the above research project

Name of investigator(s): Prof Tony Jorm, Dr Alyssia Rossetto, Ms Kathryn Chalmers, Dr Kathy Bond, Ms Shurong Lu, Ms Julia Lyons, Ms Fairlie Cottrill, Dr Claire Kelly, Dr Nicola Reavley, Ms Louise Kelly and Dr Amy Morgan.

Do you wish to participate in this survey?

If Yes, please read and sign this form and then complete the attached survey.

If No, please hand in this blank form and the survey with everyone else.

1. I consent to participate in this project, the details of which have been explained to me, and I have been provided with a written plain language statement to keep.
2. I understand that after I sign and return this consent form it will be retained by the researcher.
3. I understand that my participation will involve completion of questionnaires and I agree that the researcher may use the results as described in the plain language statement.
4. I acknowledge that:

   (a) the project is for the purpose of research;

   (b) the possible effects of participating in the questionnaires have been explained to my satisfaction;

   (c) I have been informed that I am free to withdraw from the project at any time without explanation or prejudice and to withdraw any data I have provided that has not been analysed and used;

   (d) I have been informed that the confidentiality of the information I provide will be safeguarded subject to any legal requirements.

Name of participant (Please Print): ______________________________

Participant signature: ______________________________ Date: ______________

Evaluation of the Non-Suicidal Self-Injury (NSSI) course

Investigators: Prof Tony Jorm, Dr Alyssia Rossetto, Ms Kathryn Chalmers, Dr Kathy Bond, Ms Fairlie Cottrill, Ms Shurong Lu, Ms Julia Lyons, Ms Louise Kelly, Dr Claire Kelly, Dr Nicola Reavley and Dr Amy Morgan.

What is Mental Health First Aid?

Mental health first aid is the help provided to a person developing a mental health problem, experiencing a worsening of an existing mental health problem, or in a mental health crisis. The help is given until appropriate professional help is received or until the crisis resolves. Mental Health First Aid Australia develops evidence-based training courses that teach community members mental health first aid skills. For more information, see: www.mhfa.com.au

What is the purpose of this research

The purpose of this research is to investigate the impact of the training on:

- Mental health first aid intentions and actions
- Mental health first aid knowledge
- Confidence in providing mental health first aid
- Views about non-suicidal self-injury (NSSI).

Who is conducting this research?

The Centre for Mental Health, Melbourne School of Population and Global Health at the University of Melbourne, and Mental Health First Aid Australia are conducting research to evaluate a new course about helping someone who is engaging in non-suicidal self-injury. Some of the investigators of this research are affiliated with Mental Health First Aid Australia: Tony Jorm is Chair of the Board, Kathy Bond and Fairlie Cottrill are research officers, Nataly Bovopoulos is CEO, and Fiona Blee, Louise Kelly and Claire Kelly are program managers. The data analysis component of this research will, however, be managed by members of the research team from the University of Melbourne. The contributions of each researcher, and any competing interests, will be declared in any publications resulting from the research.

What will I be asked to do?

To track if your knowledge about non-suicidal self-injury and first aid strategies change after the course, you will be asked to complete questionnaires before and after the course has finished. The questionnaires will each take around 20 minutes to complete. After six months, you will be sent an email with a link to a questionnaire similar to the pre- and post-course questionnaires. We may contact you to remind you to complete the six-month questionnaire by email or phone.

Is participation in this research confidential?

All information provided when responding to the questionnaires is confidential. None of your information or responses will be shared with anyone outside of the research team, including your employers, co-workers, etc. All results from the questionnaires will be published in the form of group percentages or averages or in a de-identified form. The information provided by participants will be stored electronically and held under password protection, for a minimum period of 5 years. The data will not be used for any purposes other than those described here.

How will the outcome/results of this research be made public?
The findings of this research will be published in a scientific journal, on the Mental Health First Aid website (www.mhfa.com.au) and in training materials for adults. Results may also be presented and discussed at conferences on mental health.

What if I change my mind?
Participation in this research is voluntary. You are able to stop participation in the questionnaires at any time. You can also withdraw your data by contacting the researchers. If you decide to withdraw from this research, all contact details will be deleted immediately.

Are there any risks?
Some people find participating in Mental Health First Aid training a little distressing because it can be difficult to think and talk about mental illness. However, most participants also report benefits from having learnt about how they can help others with a mental illness. If you are distressed about the issues discussed in the training, please talk to the instructor or you can contact one of services below.

What if I feel distressed after participating in the research?
If taking this survey brings up difficult emotions, you may wish to contact **Lifeline on 13 11 14** or **Suicide Call Back Service on 1300 659 467** for free online and telephone counselling, available 24 hours a day, seven days a week. Please also contact the Research Officer, Dr Kathy Bond on tel: 03 9079 0207 or email: kathybond@mhfa.com.au so that we can better understand how these surveys affect participants.

What are the benefits?
By undertaking the course, participants will be taught a range of skills that may be useful in assisting a person they know who engages in NSSI. The information provided in the questionnaires will assist us in developing a training package of the highest possible quality. If found to be effective, the training package will be used by the Mental Health First Aid program in the future. Participation in this research will therefore benefit future course participants across Australia and internationally, who receive the Mental Health First Aid for Non-suicidal Self-injury course. In addition, this project has the potential to lead to better community support for people who engage in NSSI.

Does this research have ethics approval?
This research project has been approved by the Human Research Ethics Committee of The University of Melbourne. If you have any concerns or complaints about the conduct of this research project, which you do not wish to discuss with the research team, you should contact the Manager, Human Research Ethics, Office for Research Ethics and Integrity, University of Melbourne, VIC 3010. Tel: +61 3 8344 2073 or Fax: +61 3 9347 6739 or Email: HumanEthics-complaints@unimelb.edu.au. All complaints will be treated confidentially. In any correspondence please provide the name of the research team and the name or ethics ID number of the research project.

How can I get further information?
If you would like further information before deciding whether to participate, please contact Dr Alyssia Rossetto, Research Manager, Mental Health First Aid Australia. Tel: +61 3 9079 0207 or email: kathybond@mhfa.com.au

If you have concerns about the scientific aspects of the study, please contact
A/Professor Nicola Reavley, University of Melbourne. Tel: +61 3 9035 7628 or
email: nreavley@unimelb.edu.au
